# Study of evoked potentials and early development in premature infants

**DOI:** 10.3389/fped.2025.1571113

**Published:** 2025-11-06

**Authors:** Kyeongil Min, Minjae Jeong, Du Hwan Kim, Byung Chan Lee, Eun Sun Lee, Hyun Iee Shin

**Affiliations:** 1 Department of Rehabilitation Medicine, College of Medicine, Chung-Ang University Hospital, Chung-Ang University, Seoul, Republic of Korea; 2 Department of Pediatrics, College of Medicine, Chung-Ang University Hospital, Chung-Ang University, Seoul, Republic of Korea

**Keywords:** evoked potential, preterm birth, gross motor function measure, neurodevelopmental outcome, neurological examination

## Abstract

**Introduction:**

Evoked potentials have been suggested as potential biomarkers for predicting neurodevelopment. This study aimed to investigate the relationship between evoked potentials (EP) and neurodevelopmental outcomes in preterm infants.

**Methods:**

Premature infants admitted to the neonatal intensive care unit of a tertiary referral hospital between March 2020 and March 2023 were included in the study. Among them, only those with EP at a corrected age of 40 weeks were included, meanwhile, infants who did not undergo the test or had abnormal results were excluded. Additionally, patients with follow-up developmental outcomes such as the Hammersmith Infant Neurological Examination (HINE) at 3 months corrected age or the Gross Motor Function Measure (GMFM) at 6 months corrected age were included.

**Results:**

A total of 24 participants were included in this study. No significant differences in the clinical factors and results of the EP studies were observed between the two groups divided by a HINE score of 60. Hierarchical logistic regression analysis revealed that visual EP was the only factor that correlated with the lying and rolling domains of the GMFM (*P* = 0.028).

**Discussion:**

A significant association was observed between the GMFM and visual EP. Integrating the visual EP latency with other parameters may improve clinical assessments to predict developmental outcomes, possibly improving the accuracy of medical interventions and patient outcomes.

## Introduction

In recent years, the incidence of preterm births has been increasing in high-income countries. The increase may be due to advances in neonatal intensive care, subsequently leading to a positive impact on the survival rates of preterm infants (1, 2) and an increase in multiple gestations along with *in vitro* fertilization (3). However, the fact that these preterm infants are at risk of a range of serious disabilities and adverse neurodevelopmental outcomes is well known (4). There is therefore a need for greater attention to methods of assessment of neurodevelopment in preterm infants.

With the development of different diagnostic techniques and assessment methods, previous studies have confirmed that the combination of imaging tests such as brain magnetic resonance imaging (MRI) and ultrasonography along with clinical assessment including Hammersmith Infant Neurological Examination (HINE) and general movement at an early age can predict the risk of cerebral palsy (CP) with an accuracy of over 90% (5). Despite the high accuracy demonstrated by previous assessment methods, there has been continued interest in the development of additional tests that are safe, practical and applicable to preterm infants.

Evoked potentials (EPs) have also been considered useful in predicting such outcomes, as confirmed by previous studies (6, 7). In some studies, visual EPs (VEPs) and somatosensory EPs (SEPs) were identified to predict outcomes in newborns with asphyxia (8, 9). An association between Auditory EPs (AEPs) and clinical apnea in preterm babies has been strongly confirmed. When AEPs conduction time was prolonged, moderate to severe apnea was observed in preterm infants; conversely, when conduction time was short, no such apnea was observed (10). EPs are classified as normal or abnormal according to their waveform characteristics and latency parameters. Abnormal VEPs reports are associated with neurodevelopmental outcome including CP (7), whereas normal SEP reports are associated with normal neurodevelopmental outcome (9). In patients with multiple sclerosis, AEPs may, at times, exhibit greater sensitivity than MRI in the assessment of brainstem function. However, MRI scans can also show high signal intensity lesions even when EP findings are normal (11). These findings suggest that it is important to consider EP latency as a continuous variable, rather than dividing it into normal and abnormal categories based on standard reference values. Therefore, this study aimed to investigate whether results without “no response” helped predict outcomes and to further investigate the relationship between EPs and neurodevelopment, focusing on the continuous relationship between EP latency and neurodevelopment. In this context, our hypothesis was that even among preterm infants whose EP results fall largely within the conventional “normal” range, subtle variations in EP latency would be significantly associated with neurodevelopmental outcomes. We further hypothesised that analysing EP measures as continuous variables, rather than dichotomising them, would allow us to detect early vulnerabilities that may not be captured by a simple normal/abnormal classification.

## Methods

### Participants

Electronic medical records and EP data of preterm infants (gestational age <37 weeks) admitted to the neonatal intensive care unit at Chung-Ang University Hospital (CAUH) between March 2020 and March 2023 were retrospectively reviewed. In CAUH, preterm infants who underwent EP assessment at approximately 40 weeks of corrected age were included. Preterm infants who did not undergo an EP assessment were excluded for reasons including other medical problems, a lack of parental consent, or transfer to another hospital. In total, 36 infants underwent EP assessment, and after excluding cases with “no response,” 30 infants were included. Of these 30 infants, 20 underwent HINE evaluation and 22 underwent GMFM assessment. Patients with a follow-up HINE assessment at 3 months of corrected age or a Gross Motor Function Measure (GMFM) at 6 months of corrected age were included in this study.

### Clinical information

Clinical information in electronic medical records of the infants during the perinatal and postnatal periods was obtained including (1) infantile characteristics: gestational age, sex, birth weight, HINE, GMFM; (2) neonatal morbidities: 1- and 5-min Apgar scores, intraventricular hemorrhage, periventricular leukomalacia, seizure, patent ductus arteriosus, respiratory distress syndrome, bronchopulmonary dysplasia, duration of invasive ventilator, retinopathy of prematurity, and history of sepsis; (3) maternal characteristics in electronic medical records: maternal pre-eclampsia, gestational diabetes mellitus, maternal chorioamnionitis and administration of prenatal steroid.

### EP assessment

EP assessment was performed in preterm infants admitted to the CAUH ICU, unless the presence of severe seizures or significant neurological impairment was contraindicated, and was performed in preterm infants at a corrected age of approximately 40 weeks. Owing to the difficulty of performance, the test was performed in the morning while the infant was sleeping to prevent movement during the procedure. The study was conducted using a NICOLET EDX SYNERGY machine (Natus Medical, Inc., Pleasanton, California), and the test was performed by a single clinical pathologist.

### SEP

The test was performed using the posterior tibial nerve SEP method. The reference electrode was placed on the midline frontal (Fz), and the recording electrode was placed on the midline central (Cz) to record the EP by applying electrical stimulation between the medial malleolus and Achilles tendon. The stimulus intensity was adjusted until a clear waveform was visible, and the stimulation was repeated three times per second for at least 30 trials (12, 13).

### AEP

For auditory stimulation, headphones were used to deliver broadband clicks at an intensity of 60–70 dB and a stimulation rate of 12 clicks per second. The reference electrode was placed on Fz, and the recording electrodes were placed on the left and right earlobes (A1 and A2, respectively) (14).

### VEP

The reference electrode was placed on the occipital electrode, and the recording electrode was placed on Cz. The flash stimuli were presented at a distance of approximately 20 cm in front of the infant's eyes. Each trial consisted of at least 30 responses, two times per second, and three trials were performed to obtain VEP values (15, 16).

### HINE assessment

HINE was assessed by a single experienced physician in an outpatient setting at a corrected age of 3 months. The HINE consists of five sections, (1) cranial nerve function, (2) posture, (3) movement, (4) tone, and (5) reflexes and reactions, with points ranging from 0 to 78. The global score at a corrected age of 3–4 months ranges from 62.5 to 69 (17). Study participants were then divided into the following two groups according to their HINE scores: HINE <60 and ≥60 (17).

### GMFM assessment

The GMFM was assessed by a single physiotherapist in an outpatient setting at the corrected age of 6 months. The GMFM-88 was applied in our study and consists of five assessment sections: (1) lying and rolling, (2) sitting, (3) crawling and kneeling, (4) standing, and (5) walking, running, and jumping (18). Certain assessment items in the GMFM-88 were not applicable for scoring in our study because of the developmental process at a corrected age of 6–7 months. In addition, lower scores were often obtained in the second section. Therefore, as the main target of the assessment, we focused on the first section, “lying and rolling.”

### Statistical analysis

The latency of EPs was compared between infants with HINE < 60 and those with HINE ≥ 60 using the Student's *t*-test or Mann–Whitney *U*-test. Multiple hierarchical regression analyses were performed for all participants, with the lying and rolling score domains of the GMFM as the dependent variables. In step one, maternal demographic and clinical characteristics, including maternal pre-eclampsia, gestational diabetes mellitus, and multiple gestations, were included as independent variables. Furthermore, infant characteristics such as birth weight, gestational age, and sex were included in step two. Step three extended the analysis by adding neonatal morbidities, including a history of sepsis, periventricular leukomalacia, seizures, respiratory distress syndrome, bronchopulmonary dysplasia, and retinopathy of prematurity to those included in step two. In step four, additional variables obtained from the EPs were added to the factors considered in step three. All statistical analyses were performed using IBM SPSS 24 (IBM@SPSS, Armonk, NY, USA), and statistical significance was set at *P* < 0.05.

## Results

### Study participants

**Figure 1:**
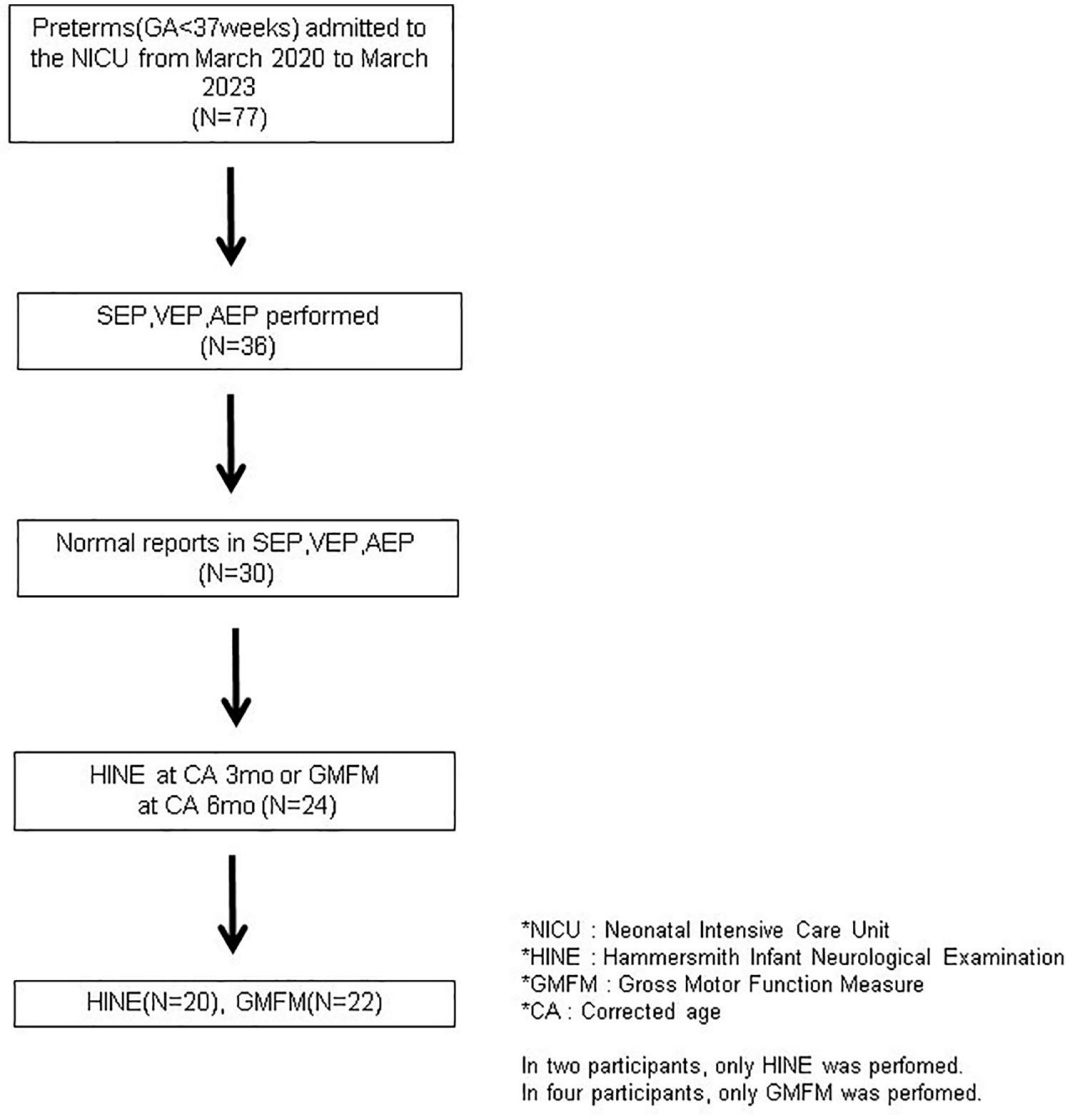
Overview of the participant selection and recruitment procedure.

Thirty participants were initially included in the study. Among them, six individuals were excluded because they did not undergo the GMFM and HINE assessments at the study time point. Consequently, 20 participants underwent the HINE assessment, while 22 participants underwent the GMFM assessment (Figure 1).

### Baseline characteristics of the participants

The baseline clinical characteristics of the study participants are displayed in Table 1. The study population consisted of 17 females (70.8%). The mean gestational age of all participants was 28 weeks and 4 days, with a mean birth weight of 1,086.2 g. Additionally, EP assesment was performed at an average corrected age of 40 weeks and 2 days. The analysis focused primarily on the latency of left-sided EPs in the current study. This decision was based on the observation that the latency of the left-hemisphere EPs tended to be faster than the right-hemisphere. No significant differences in the clinical characteristics were observed between the HINE > 60 and HINE < 60 groups.

**Table 1:** Baseline characteristics of the participants.

Clinical factor / statistical value			
	Total (*n* = 24)	Total (*n* = 20)	
		HINE < 60(*n* = 10)	HINE ≥ 60 (*n* = 10)
Infantile characteristic			
Gestational age (weeks:days)	28:4.12 ± 3:1.41	28:1.20 ± 3:5.14	29:1.30 ± 2:5.92
Corrected age at EP study (weeks:days)	40:2	40:2	40:2
Birth weigh (g)	1,086.21 ± 163.9	960.2 ± 236.52	1,201.9 ± 284.23
Global score of HINE		53.2 ± 3.86	65.6 ± 2.32
Corrected age at HINE (weeks:days)		59:2	55:3
Sex			
Male	7 (29)	4 (40)	2 (20)
Female	17 (71)	6 (60)	8 (80)
Neonatal morbidities			
APGAR scroe 1 min	3.71 ± 0.69	3.6 ± 1.21	4 ± 1.09
APGAR scroe 5 min	6.46 ± 0.59	6 ± 0.88	6.9 ± 0.99
Intraventricular hemorrhage	4 (16.7)	1 (10)	2 (20)
Periventricular leucomalacia	3 (12.5)	1 (10)	0
Seizure	2 (8.3)	1 (10)	0
PDA	21 (87.5)	8 (80)	10 (100)
RDS	18 (75)	9 (90)	6 (60)
BPD	16 (66.7)	6 (60)	3 (30)
Duration of invasive ventilator (days)	16.13 ± 6.73	23.73 ± 11.63	10.5 ± 6.28
ROP	6 (33.3)	5 (50)	2 (20)
Sepsis	3 (12.5)	1 (10)	0
Maternal factor			
Preeclampsia	5 (20.8)	3 (30)	1 (10)
GDM	1 (2.1)	0	1 (10)
Chorioamnionitis	6 (25)	3 (30)	3 (30)
Prenatal steroid	22(91.7)	10(100)	9(90)

Data are presented as the mean ± SD or *n* (%). Total participants (*n* = 24) are those who had at least one assessment, either HINE (Hammersmith Infant Neurological Examination) at corrected age 3 months or GMFM (Gross Motor Function Measure) at corrected age 6 months.

EP, evoked potential; HINE, Hammersmith infant neurological examination; GDM, gestational diabetes mellitus; RDS, respiratory distress syndrome; BPD, bronchopulmonary dysplasia; ROP, retinopathy of prematurity; PDA, patent ductus arteriosus.

### Comparisons evoked potential latencies of HINE < 60 and HINE ≥ 60 groups

The EP latencies were compared between the two groups based on a HINE score of 60 (Table 2). No significant differences in any of the EP measurements, including SEP, AEP, or VEPs, between the two groups were observed.

**Table 2:** Comparisons evoked potential latencies of HINE < 60 and HINE ≥ 60 groups.

EP wave form / statistical value				
	Total (*n* = 20)			
		HINE < 60 (*n* = 10)	HINE ≥ 60(*n* = 10)	*P*-value
SEP P0	30.98 ± 1.48	31.93 ± 2.2	29.93 ± 1.83	0.195
SEP P1	36.19 ± 1.43	37.04 ± 2.09	35.23 ± 1.84	0.224
SEP N1	44.88 ± 2.04	44.88 ± 2.86	44.89 ± 3.09	0.996
AEP 1	1.59 ± 0.1	1.54 ± 0.1	1.64 ± 0.18	0.353
AEP 3	4.33 ± 0.2	4.12 ± 0.13	4.53 ± 0.34	0.052
AEP 5	6.15 ± 0.15	6.14 ± 0.11	6.16 ± 0.282	0.909
VEP N2	60.68 ± 6.09	61.58 ± 10.11	59.78 ± 7.31	0.781
VEP P2	102.97 ± 3.02	102.54 ± 5.5	103.39 ± 2.86	0.792
VEP N3	146.03 ± 4.35	146.71 ± 6.31	145.34 ± 6.3	0.767

Data are presented as the mean ± SD.

SEP, somatosensory evoked potential; AEP, auditory evoked potential; VEP, visual evoked potential; SEP P0, the first significant positive deflection in the waveform; SEP P1, a positive peak in the waveform after P1; SEP N1, a negative peak in the waveform.

### Hierarchical logistic regression analysis of the lying and rolling domain of GMFM

In the hierarchical logistic regression analysis (Table 3), only the VEP exhibited a significant relationship with the lying and rolling domains of the GMFM (*P* = 0.028). The R^2^ value in step four was 0.932, indicating a high explanatory power of 93.2% for the lying and rolling domains of the GMFM. Furthermore, significant associations were identified for VEP P2 (*P* = 0.005) and VEP N3 (*P* = 0.023) when examining the association of each independent variable in step four. Except for sex (*P* = 0.012), no variables displayed significant associations.

**Table 3:** Hierarchical logistic regression analysis of lying & rolling domain of GMFM (gross motor function measure).

Clinical factor & EP wave / statistical value						
	*B*	SE	*β*	*t*	*p*	*R* ^2^
Step 1	40.7	3.165		12.860	0.00	0.102
PE	4.7	5.481	0.206	0.857	0.402	
GDM	10.3	10.496	0.225	0.981	0.339	
Multiple	5.8	5.168	0.27	1.122	0.276	
Step 2	9.798	27.972		0.35	0.731	0.352
PE	−0.253	6.348	−0.011	−0.04	0.969	
GDM	13.230	9.950	0.289	1.33	0.203	
Multiple	6.462	5.035	0.301	1.283	0.219	
BW	−0.005	0.008	−0.195	−0.556	0.586	
GAd	0.210	0.175	0.475	1.200	0.249	
Sex	−8.184	4.459	−0.339	−1.836	0.086	
Step3	41.811	45.019		0.929	0.377	0.578
PE	−0.031	10.808	−0.001	−0.003	0.998	
GDM	16.637	13.934	0.363	1.194	0.263	
Multiple	3.995	9.492	0.186	0.421	0.684	
BW	−0.008	0.01	−0.353	−0.799	0.445	
GAd	0.097	0.239	0.221	0.407	0.694	
Sex	−8.752	7.285	−0.427	−1.201	0.26	
Sepsis	11.318	12.109	0.341	0.935	0.374	
PVL	3.893	12.571	0.14	0.31	0.764	
Seizure	−9.791	11.964	−0.295	0.818	0.434	
RDS	−0.245	9.935	−0.011	−0.025	0.981	
BPD	−3.146	6.507	−0.384	−0.484	0.640	
ROP	−2.916	8.429	−0.142	−0.346	0.737	
Step4	89.586	31.1		2.881	0.028*	0.932
PE	5.426	5.591	0.238	0.971	0.369	
GDM	12.394	6.932	0.270	1.788	0.124	
Multiple	10.189	4.808	0.475	2.119	0.078	
BW	−0.004	0.007	−0.184	−0.623	0.556	
GAd	0.04	0.122	0.09	0.326	0.755	
Sex	−13.571	3.815	−0.662	−3.557	0.012*	
Sepsis	−11.442	7.886	−0.045	−0.223	0.831	
RDS	−7.41	6.17	−0.345	−1.201	0.275	
BPD	1.848	3.343	0.225	0.553	0.6	
ROP	−8.457	4.382	−0.412	−1.93	0.102	
VEP N2	−0.031	0.154	−0.051	−0.199	0.849	
VEP P2	−1.395	0.321	−1.653	−4.345	0.005*	
VEP N3	0.738	0.240	1.177	3.038	0.023*	

PE, preeclampsia; GDM, gestational diabetes mellitus; BW, birth weigh; GAd, Gestational age (day); RDS, respiratory distress syndrome; BPD, bronchopulmonary dysplasia; ROP, retinopathy of prematurity; VEP, visual evoked potential.

* Statistical significant values, *p* < 0.05.

## Discussion

In this study, VEP demonstrated a strong correlation with GMFM at 6 months of corrected age, with the significance remaining high when compared with other clinical characteristics using hierarchical regression analysis. However, the association between the HINE results and EPs has not been confirmed.

In the hierarchical logistic regression analysis, VEP had significant associations that were distinct from those of AEP and SEP, particularly with GMFM scores, compared to other factors. The timing of synaptogenesis in different brain areas may account for their unique association with VEP. According to a study by Huttenlocher and Dabholkar on the maturation of the brain, the visual areas reach their peak of synaptogenesis earlier than the auditory and sensory areas (19). Therefore, these results suggest that the observed associations, particularly in VEP, may be due to the different maturation processes that occur in different regions of the brain. The aforementioned findings are consistent with the results of a previous study that established that the auditory and visual areas of the brain develop more rapidly than the sensory areas during the first year after birth (20). This study demonstrated that the EPs can be a valuable diagnostic technique for the assessment of neurodevelopment in preterm and term infants, which is in line with previous research. This highlights the usefulness of EPs in assessment and reaffirms the association between EPs and neurodevelopmental outcomes (21).

This study only included preterm infants with “normal VEP reports.” Previous studies that compared VEP with neurodevelopmental outcomes displayed that VEP was very useful in predicting neurodevelopmental outcomes in preterm infants, with a specificity of 94% but a relatively lower sensitivity of 78% (22). This suggests that the possibility of neurological deficits could not be completely excluded, even in cases where the VEP reports were within the normal range. Therefore, in this study, significant associations between latency differences within the normal range of VEP reports and neurodevelopmental outcomes were confirmed using hierarchical logistic regression analysis. This highlights the need for continuous interpretation of VEP reports rather than binary normal/abnormal interpretations. In addition, the P2 component of the VEP showed a negative correlation with the lying and rolling domain of the GMFM in the hierarchical logistic regression analysis. Conversely, the N3 component demonstrated a positive correlation (Table 3). This suggests that the latency of the P2 component is not delayed as the score of the lying and rolling domain of the GMFM increases, whereas, in contrast, the N3 component appears to be increasingly delayed. According to previous studies, the N3 component is associated with the sleep-wake cycle and has a longer latency during sleep (23). In the present study, EP assessments were conducted while the preterm infants were asleep. This may have influenced the N3 component. Therefore, the P2 component, which is unaffected by the sleep-wake cycle, may be considered a more reliable indicator.

Although no significant differences in the EP latencies between the two study groups divided by HINE scores were identified, noting that the study's limited sample size and the early timing of HINE assessment may have contributed to these results is important. Therefore, to draw definitive conclusions regarding the prediction of developmental status at 3 months using EPs based on the results obtained in this study is difficult. The *P*-value for AEP wave three was 0.052, indicating a relatively closer statistical significance compared with the other SEP and VEP measures. A previous study by Wang et al. demonstrated a strong association between AEP and neurodevelopmental outcomes in preterm infants using the Bayley Scale (24). Although this study did not demonstrate a statistically significant correlation between AEP and HINE, previous studies have reported significant associations between AEP and the Bayley Scales. Therefore, the potential importance of conducting EP studies within the first three months of life in preterm infants should not be underestimated.

In this study, the focus of the analysis was on the values of the left-hemisphere EPs. This decision was based on the observation that in the actual data from the participants, the measurements obtained from the left hemisphere generally demonstrated faster responses than those from the right hemisphere. Differences between the left and right hemispheres have been observed in neuroimaging techniques such as Neurite Orientation Dispersion and Density Imaging or Neurite Orientation Dispersion (25). This indicates that asymmetry exists in brain development between the left and right hemispheres. Furthermore, other studies in healthy adults have confirmed a significant difference in amplitude between the left and right hemispheres in VEP testing (26, 27). Therefore, the EP results exhibiting differences between the left and right hemispheres, as observed in this study, suggest that such asymmetry was not unique to this study particularly, but rather a common feature.

The limitation of this study are the small sample size and retrospective nature, which may have led to a lack of power in the analysis. However, as the participants in this study are at high risk and the incidence itself is low, it could be considered that the sample size is enough given this situation in South Korea (28). Another limitation of this study is that the follow-up period for evaluating neurological outcomes following the EP study was relatively short at only a few weeks. Moreover, incorporating parental socioeconomic status into the analysis might have helped to better explore its potential association with the EP study (29). The value of this study is significant given the inherent difficulties in conducting research specifically on preterm births. The results of this study should be re-evaluated in future studies that have large sample sizes and long-term follow-ups, and also are prospective in nature. This is an important step in validating and extending the results of this study.

In conclusion, by combining VEP latency with other relevant factors, clinicians may increase the accuracy of their predictions. This integrated approach has the potential to improve the precision of clinical interventions, aid in the early detection of neurodevelopmental abnormalities in preterm infants and ultimately reduce the risk of long-term neurological damage and associated complications through timely and proactive management.

## Data Availability

The raw data supporting the conclusions of this article will be made available by the authors, without undue reservation.
